# Associations between body composition and cardiovascular disease risk in pre- and postmenopausal women

**DOI:** 10.1186/s41043-023-00455-6

**Published:** 2023-10-17

**Authors:** Nirmala Rathnayake, Gayani Alwis, Janaka Lenora, Sarath Lekamwasam

**Affiliations:** 1 Department of Nursing, Faculty of Allied Health Sciences, University of Ruhuna, Matara, Sri Lanka; 2 Department of Anatomy, Faculty of Medicine, University of Ruhuna, Matara, Sri Lanka; 3 Department of Physiology, Faculty of Medicine, University of Ruhuna, Matara, Sri Lanka; 4 Population Health Research Centre, Department of Medicine, Faculty of Medicine, University of Ruhuna, Matara, Sri Lanka

**Keywords:** Associations, Body composition, Cardiovascular disease risk, Premenopausal women, Postmenopausal women

## Abstract

**Background:**

Menopause transition is a critical phase of women’s life since body composition and cardiovascular risk factors begin to change during this period. This study investigated the associations between body composition and cardiovascular disease risk (CVDR) in pre (PrMW) and postmenopausal women (PMW).

**Methods:**

A community-based cross-sectional study involving 184 PrMW and 166 PMW, selected randomly from Bope-Poddala area in Galle, Sri Lanka was carried out. Total-body fat mass (TBFM, kg), total body skeletal muscle mass (TBSMM, kg), total body bone mineral density (TBBMD, g/cm^2^) and total body bone mineral content (TBBMC, g) were measured with total body DXA scanner and they were taken as indices of body composition. CVDR was evaluated using Framingham risk score (FRS%) and individual CVDR factors, such as systolic blood pressure (SBP, mmHg), diastolic blood pressure (DBP, mmHg), fasting blood sugar (FBS, mg/dl), total cholesterol, (TC, mg/dl), tryglycerides (TG, mg/dl), high-density lipoprotein (HDL, mg/dl) and low-density lipoprotein (LDL, mg/dl). Correlations between indices of body composition and CVDR factors were assessed with adjusted partial correlation (adjusted for socio-demographic and gynecologic status, age, daily calorie consumption and physical activity level).

**Results:**

Mean(SD) age of PrMW and PMW were 42.4(6.0) and 55.8(3.8) years respectively. TBFM correlated with SBP and DBP (*r* range; 0.15 to 0.21) and TBSMM correlated with SBP, DBP and HDL (*r* range; − 0.24 to 0.17) only in PrMW (*p* < 0.05). TBBMD correlated only with FBS in PMW (*r*; − 0.21, *p* = 0.01). TBBMC did not show correlations with CVDR factors (*p* > 0.05). Body composition indices did not show correlations with total CVDR estimated by FRS and in both groups of women (*p* > 0.05).

**Conclusions:**

Both SBP and DBP are associated with FM and SMM in different ways among PrMW. This association, however, was not seen among PMW. FBS is associated with BMD only in PMW.

## Background

Menopause is a critical phase of women’s life as the onset of cardiovascular disease (CVD), obesity, osteoporosis and sarcopenia is directly linked with menopause transition [1]. The prevalence of CVDs in developing countries is increasing alarmingly [2]. Although considered a disease predominantly seen in men, CVD is becoming prevalent among women as well. The incidence of CVD is higher among postmenopausal women (PMW) compared to premenopausal women (PrMW), even after adjusting for risk factors [3]. Studies have shown that CVD is one of the leading causes of death in PMW [4]. The incidence of CVD in middle aged women increases dramatically after menopause, probably due to direct and indirect effects of low serum estrogen [5].

Accumulation of fat mass (FM), both total and regional [6], and loss of skeletal muscle mass (SMM) [7] is seen with advancing age and this is partly due to hormonal depletion associated with menopause [6, 7]. Kim et al. found that increased FM and reduced SMM lead to obesity and sarcopenia, respectively, and these have close associations with insulin resistance, dyslipidemia, hypertension, metabolic syndrome and all-cause mortality [8]. Low bone mineral density (BMD) and bone mineral content (BMC) that are predominantly seen after menopause have also shown associations with surrogate markers of CVD [9, 10]. Tanko et al. observed that PMW with osteoporosis have an increased risk of acute cardiovascular (CV) events independent of age and cardiovascular risk profile, and the CV risk is proportional to the severity of osteoporosis at the time of the diagnosis [11]. Furthermore, PMW with low BMD have lipid profile that promotes atherosclerosis [11, 12]. Unlike FM, relationships between CVD risk (CVDR) and SMM or BMC are uncertain as studies have shown inconsistent results [13–15]. The data related to the body composition and CVDR are limited in local context. Due to the limited research data, the exact relationship between CVDR and different body components during menopausal transition among Sri Lankan women is not fully understood. Most of the studies in this area have been done in Western and South-East Asian countries and there is a scarcity of research in South Asian countries including Sri Lanka. Relationships between CVDR and body composition among Sri Lankan women may be different from those seen among their counterparts in western countries, because of well-known genetic and other variations in body composition and CVDR factors in different geographical regions [16–18]. Therefore, direct applicability of data originating from western populations to Sri Lankan population is questionable. Studies in this area will help the health promotion activities to promote both body composition and cardiovascular health at optimum among women in future. Hence, the current study was designed to investigate the associations between body composition and CVDR in a group of PrMW and PMW selected from Sri Lanka.

## Methods

### Study design, setting and sample

A community-based cross-sectional study was carried out in the community study area of the Faculty of Medicine, University of Ruhuna in Galle district, Sri Lanka, during June 2015 to January 2017. This study was designed as a part of a research project titled “Effects of menopause on bodily structure, functions and physical health”. The data were collected at the Research Laboratory of Department of Physiology, Faculty of Medicine, University of Ruhuna by the principal investigator under optimum conditions.

Healthy community-dwelling PrMW (*n* = 184) and PMW (*n* = 166), aged 30–60 years, selected based on the multi-stage cluster sampling technique participated in the study. Out of the eighteen Public Health Midwives (PHM) divisions (the smallest community health provision area) in this community study area, 05 PHM divisions were randomly selected. The selected PHM divisions were considered as clusters. Women belonging to age 30–60 years were identified using the household registers of particular areas maintained by the government. The target was to achieve 38 PrMW and 38 PMW from each PHM division (to achieve 190 PrMW and the same number of PMW). Despite many practical issues encountered during data collection, of the desired sample sizes (190 in each group), 97% in PrMW (184) and 87% (166) in PMW were achieved.

Menopausal status was determined based on the classification of Stages of Reproductive Aging Workshop (STRAW) [19]. Women who used thyroxin, corticosteroids, insulin, hormone replacement therapy or hormonal contraceptives were excluded from the study. Those who were pregnant or lactating, on dedicated dietary programs or supervised exercise programs and those with chronic disease; non-communicable diseases (NCDs), chronic infections, polycystic ovary syndrome or chronic major organ disease were also excluded.

### Data collection and measurements

Body composition; total-body FM (TBFM, kg), total-body SMM (TBSMM, kg), total-body BMD (BMD, g/cm^2^) and total-body BMC (TBBMC, g) were measured with a total body central-type DXA scanner (Hologic Discovery W, Hologic Inc, Bedford, MA, USA) adhering to the manufacturer’s protocol. Apart from that, trunkal FM (TrFM, kg), total hip BMD (THBMD, g/cm^2^) and appendicular SMM (ASMM, kg) were also measured. The procedure was carried out by the same technician who calibrated the device each scanning day. Analytical software provided by the DXA manufacturer was used to analyze body composition.

Socio-demographic information were collected using a pre-designed questionnaire. Physical activity (PA) level was evaluated with short version of international PA questionnaire (IPAQ), which was translated in to Sinhala language and pre-tested. In the IPAQ, participants were asked to report the time on walking, moderate intensity activity, vigorous intensity activity during the week prior to the interview to calculate the total PA score. The PA data were converted to minute per week and expressed as a metabolic equivalent (MET-min/week) according to the IPAQ guidelines for data processing [20].

Daily total energy consumption (kcal/day) was obtained from a 24-h dietary recall (HDR) method. The subjects were asked to recall all foods and beverages, consumed over the previous 24-h period. Respondents were probed for the types of foods and food preparation methods. For uncommon mixed meals, the details of recipes and preparation methods were collected at the time of taking the 24 HDR. In addition, a detailed description of the foods (brand names of some foods, such as milk and processed foods) was recorded. As dietary assessment aids, the standard household measurements such as plate, bowl, cup, glass and different spoons were used to facilitate the quantification of portion sizes. One medium-sized coconut spoon of rice was taken as 100 g, a full plate as 400 g, one cup of liquid as 150 ml, one glass of liquid as 200 ml, a table spoon as 15 g and a tea spoon was taken as 5 g. Household measurements were clarified by demonstration of the real utensils. For different curries, weights of average respective amounts were taken. All foods recorded in 24 HDR were converted into grams and when subjects recalled some food amount in grams, that information was directly obtained. Then, the intake of total energy were analyzed using Indian food composition table [21] and Sri Lankan food composition tables [22].

CVDR was evaluated with individual CVDR factors; blood pressure (BP); systolic blood pressure (SBP, mmHg), diastolic blood pressure (DBP, mmHg), fasting blood sugar (FBS, mg/dl), lipid profile; total cholesterol (TC, mg/dl), triglycerides (TG, mg/dl), high-density lipoproteins (HDL, mg/dl) and low-density lipoprotein (LDL, mg/dl) and future 10 year CVDR; Framingham Risk Score (FRS %).

SBP and DBP were recorded on the right arm in the seated position after the subject has rested for 15 min using a sphygmomanometer (BOKANG Instrument Co. Ltd, China, CEO 197), twice with 15 min interval between the measurements. Mean of two trails was obtained.

A venous blood sample of 4 ml was drawn from the anti-cubital vein in the non-dominant side in the morning after the subject had fasted for 10–12 h. Serum samples were assessed for FBS, TC TG and HDL using Mindray (BA–88A) semi auto chemistry analyzer (China) with the chemical reagents recommended for each assays. All the investigations were performed as duplicate tests at the standard laboratory premises of the Department of Medicine and Nuclear Medicine Unit, Faculty of Medicine, University of Ruhuna under expert scientific involvement.

LDL was calculated using following formula after measuring the TC, HDL and TG levels; LDL = TC − (HDL + TG/5) [23].

FRS (%) was calculated, that is a gender-specific algorithm used to estimate the 10-year CVDR of an individual. The variables that were incorporated for the calculation of FRS include age, TC, smoking status, HDL, SBP, antihypertensive medication use, and diabetes status [24].

### Statistical analyses

Descriptive statistics; means (SD) or frequency (%), were used to describe the data. Difference of evaluated variables between PrMW and PMW were compared using independent sample *t* test.

Correlations between body composition indices and CVDR factors were determined by adjusted partial correlation after controlling the possible confounding factors (sociodemographic and gynecologic status, age, calorie consumption and PA level). Data were analysed using SPSS 20.0 and *p* value < 0.05 was considered statistically significant.

### Ethical considerations

Ethical clearance for this study was obtained from ethics review committee, Faculty of Medicine, University of Ruhuna, Sri Lanka (Reference number; 24.09.2014:3.2). Written informed consent was obtained from each participants and they were interviewed and examined to obtain relevant demographic and health information.

## Results

Mean(SD) age of PrMW and PMW in the study sample were 42.4(6.0) and 55.8(3.8) years, respectively. The majority of participants in both groups were Sinhalese, unemployed, married and educated up to secondary level education or beyond (Table 1).

**Table 1:** Sociodemographic characteristics of PrMW and PMW (*n* = 350)

Characteristics	Sub category	Mean (SD) or frequency (%) PrMW (*n* = 184)	Mean (SD) or frequency (%) PMW (*n* = 166)	*p* value*
Age (years)		42.4(6.0)	55.8(3.8)	< 0.001^a^
Ethnicity	Sinhala	171 (92.9)	160(96.4)	0.11^b^
	Non Sinhala	13 (7.1)	6(3.6)	
Employment status	Employed	58(31.5)	49(29.3)	0.38^b^
	Unemployed	126(68.5)	118(70.7)	
Civil status	Married	169(91.8)	125(74.9)	< 0.001^b^
	Single or widowed or divorced	15(8.2)	42(25.1)	
Living companion	With husband and children	134(72.8)	103(61.6)	0.003^b^
	With Husband or Children	16(8.6)	35(21.0)	
	Alone or living with others	34(18.5)	29(17.4)	
Education status	Primary education	37(20.1)	46(27.6)	0.12^b^
	Secondary education	68(37.0)	64(38.3)	
	Upper secondary or tertiary education	79(42.9)	57(34.1)	
Monthly income	Below 20,000 LKR	92(50.0)	125(74.8)	< 0.001^b^
	Above 20,000 LKR	92(50.0)	42(25.2)	

LKR = Sri Lankan rupees (190LKR = 1USD), PrMW = premenopausal women, PMW = postmenopausal women

Living with others include; parents, siblings, friends or other relatives

*p values derived with groups comparison by independent sample *t* test (a) and chi square test of independence (b)

The characteristics of subjects including body composition indices and CVDR factors are shown in Table 2. PMW had higher CVDR determined by individual risk factors and overall risk score, and lower BMD and BMC and SMM compared to PrMW.

**Table 2:** Basic characteristics of PrMW and PMW (*n* = 350)

Characteristics	PrMW (*n* = 184) Mean (SD)	PMW (*n* = 166) Mean (SD)	*p* value*
Age at menopause (years)	–	48.2(3.9)	–
Time since menopause (years)	–	7.5(4.9)	–
*CVDR factors*			
SBP (mmHg)	111.7(15.0)	121.1(18.7)	< 0.001
DBP (mmHg)	74.7(10.0)	78.3(10.6)	0.001
FBS (mg/dl)	85.5(18.3)	94.7(38.3)	0.006
TC (mg/dl)	173.1(34.5)	176.7(39.8)	0.36
TG (mg/dl)	95.6(50.9)	91.4(48.0)	0.42
HDL (mg/dl)	53.5(15.0)	66.0(33.4)	< 0.001
LDL (mg/dl)	102.0(32.2)	105.3(36.7)	0.38
Overall 10 year CVDR (FRS %)	2.0(1.3)	4.2(2.2)	< 0.001
*Body composition indices*			
TBFM (kg)	18.4(5.6)	19.6(6.4)	0.30
TrFM (kg)	7.8(3.7)	8.5(2.5)	0.06
TBSMM (kg)	32.5(4.4)	30.5(5.2)	< 0.001
ASMM (kg)	16.06(2.51)	14.87(2.96)	< 0.001
TBBMD (g/cm^2^)	0.892(0.060)	0.812(0.081)	< 0.001
THBMD (g/cm^2^)	0.897(0.118)	0.831(0.121)	< 0.001
TBBMC (g)	1283.6(183.1)	1092.8(250.2)	< 0.001
*Lifestyle factors*			
Total PA score (MET/min/week)	7482.5(2400.0)	7648.0(2534.6)	0.53
Daily total energy consumption (kcal/day)	1368.2(412.5)	1154.1(331.8)	< 0.001

PrMW = premenopausal women, PMW = postmenopausal women, SBP = systolic blood pressure, DPB = diastolic blood pressure, FBS = fasting blood sugar, HDL = high density lipoproteins, LDL = low density lipoproteins, HOMA IR = homeostasis model assessment, FRS = Framingham Risk Score, CVDR = cardiovascular disease risk, TBFM = total body fat mass, TrFM = trunkal fat mass, TBSMM = total body skeletal muscle mass, ASMM = appendicular skeletal muscle mass, TBBMD = total body bone mineral density, THBMD = total hip bone mineral density, TBBMC = total body bone mineral content

*p values derived with the group comparison by independent sample *t* test

Correlations between body composition and CVDR are shown in Table 3. In PrMW, SBP and DPB showed positive correlations with TBFM and negative correlations with TBSMM. In addition TBSMM of PrMW showed a positive correlation with HDL (Table 3). The associations observed between CVDR and TrFM were similar to those seen with TBFM while the association between CVDR and ASMM was also similar to those seen with TBSMM (data not shown). Among PMW, only FBS showed an inverse correlation with TBBMD (Table 3), as well as with THBMD (data not shown). No significant correlations were observed between total CVDR estimated by FRS and body composition indices in both groups of women.

**Table 3:** Association between body composition indices and CVDR factors in PrMW and PMW (*n* = 350)

CVDR factor	TBFM	TBSMM	TBBMD	TBBMC
***PrMW***				
SBP	0.15*	− 0.23**	− 0.01	− 0.01
DBP	0.21**	− 0.24**	− 0.01	− 0.01
FBS	0.02	− 0.02	− 0.08	− 0.03
TC	0.04	− 0.04	− 0.06	− 0.07
TG	0.06	− 0.01	− 0.02	− 0.02
HDL	− 0.08	0.17*	0.01	0.09
LDL	0.006	− 0.02	− 0.007	− 0.03
FRS	0.11	− 0.14	− 0.07	− 0.06
***PMW***				
SBP	0.01	− 0.14	− 0.004	− 0.01
DBP	0.02	− 0.08	− 0.004	− 0.04
FBS	0.06	− 0.01	− 0.21**	− 0.01
TC	0.07	− 0.06	− 0.07	− 0.06
TG	0.005	− 0.10	− 0.01	− 0.08
HDL	− 0.09	0.03	− 0.10	0.04
LDL	0.04	− 0.04	0.10	− 0.09
FRS	0.001	− 0.05	− 0.04	− 0.005

TBFM = total body fat mass, TSMM = total body skeletal muscle mass, TBBMD = total body bone mineral density, TBBMC = total body bone mineral content, SBP = systolic blood pressure, DBP = diastolic blood pressure, HDL = high density lipoprotein, LDL = low density lipoprotein, FBS = fasting blood sugar, TC = Total cholesterol, TG = Triglycerides CVDR = cardiovascular disease risk, FRS = Framingham Risk Score

Adjusted partial correlations are significant at < 0.05* and < 0.01** levels

Other variables did not have significant correlations (*p* > 0.05)

## Discussion

The current study found both systolic and diastolic BPs to be positively related to FM and inversely related to SMM in PrMW. Further SMM in PrMW showed a positive association with serum HDL. Among PMW, only significant finding was the association between FBS and BMD.

Keeping with our observation, Vasiri et al. [25] and Han et al. [26] have identified lower SMM as a predictor of hypertension in young women [25, 26]. Subjects with low SMM are likely to have functional impairment and low PA which may cause a reduction in myokines which are muscle contraction‐induced factors that have anti‐inflammatory effects [27]. The relative paucity of myokines increases the CVDR, including hypertension [28]. Furthermore, the association between SMM and BP may be related to underlying vitamin D status. Those with hypovitaminosis D and low PA are likely to have low SMM and [29] high BP [30]. In addition, an association has been observed between low SMM and dysglycaemia in young women by Srikanthan and Karlmangala [31] and in our study, we were unable to observe such association. Since, the SMM is a major metabolically active compartment which accounts for 85% of whole body insulin mediated glucose disposal, high SMM enhances peripheral insulin sensitivity and improve systemic glucose homeostasis [32]. The association between SMM and HDL is still unexplainable with the available evidence.

The positive association between FM and BP in PrMW seen in this study and also in previous studies [33–35], could be mediated through PA and dietary factors. Dua et al. [33] and San et al. [35] identified that FM is positively correlated with BP while Valetino et al. [34] found that FM is closely linked with clustering of CVDR factors. The sedentary lifestyle and improper dietary practices [36] commonly seen among young women [37] would lead to high FM and high BP among them. Increased FM is associated with an increase in arterial stiffness due to negative changes in the vascular structure [38], reduced vascular elasticity due to an increase in intravascular inflammation, and decrease in the arterial luminal diameter that would contribute to high BP [38]. Further, higher body fat content enhances the sympathetic tone, activation of the renin-angiotensin system, hyperinsulinemia, and secretion of adipokines such as leptin [39] and all these derangements can potentially lead to high BP. Apart from BP, these metabolic derangements would lead to high lipids and blood glucose [34, 40] and eventually CVDR [41]. We however did not find such associations extending beyond BP.

Associations found in previous studies between BMD/BMC and CVDR factors among young women have been inconsistent. Similar to our observations, Lekamwasam et al. found associations between CVDR and BMD/BMC have not been observed among PrMW in Sri Lanka [42]. However, Saoji et al. [43] and Makovey et al. [44] have shown BMD/BMC in young women to be associated with serum lipids [43, 44] while Jeon et al. [45] and Farhat and Cauley [46] demonstrated the association between BMD/BMC with BP [45, 46].

Number of studies have observed significant associations between CVDR and high FM [7, 8, 47], and low SMM [7, 8, 48] in PMW. Chen et al. found the close association between high FM and CVDR in PMW with normal BMI [47] and Korean National Health and Nutritional Examination Surveys reported low SMM is related to the BP, lipids and blood sugar and overall metabolic syndrome [48]. PMW have increased fat deposition in the central (upper body) region [49–51] and diminished motor units in muscle fibers specially the type II fast glycolytic fibers along with loss of SMM and these changes are thought to be partly due to low estrogen level [52, 53]. These changes increase the CVDR and disrupt the systemic glucose homeostasis [32]. The exclusion of women with confirmed NCDs who have high CVDR might be a reason for not observing these associations among our study subjects. Furthermore, we did not observe associations between low BMD and lipid profile or BP although previous studies have shown such associations as shown by Bagger et al. [15] and Cuppuccio et al. [54].

Our observation between FBS and TBBMD, however, is concordant with a previous study [55] showing similar associations. This solitary correlation seen between TBBMD and FBS in our study could either be biological or due to chance. The possibility of this being a chance finding is high since this relationship was observed during a multiple comparison and no relationship was observed between TBBMC and FBS. Many studies have shown links between diabetes and osteoporosis and also between the two metabolic pathways [56, 57].

The findings of the current study indicate that young women with high FM and low SMM are at higher risk for CVD. It is well-known that SMM has a role in inflammation, contributing to energy homeostasis and the pathogenesis of obesity, type 2 diabetes mellitus, and other CVD. Additionally, SMM is the primary reservoir for amino acids to maintain protein synthesis in vital tissues and organs. Muscular strength has also been recognized in the pathogenesis and prevention of chronic diseases, due to inverse association with adiposity gains as well as risk of hypertension, T2DM and prevalence and incidence of metabolic syndrome [58]. Further, obesity characterized by high FM is linked with chronic low-grade inflammation and dysregulation of the endocrine and immune milieu in the adipose tissue. Aberrant production of adipokines and inflammatory molecules have been associated with the genesis of CVD [59].

These findings, although not new, strengthen the healthy lifestyle recommendations made at community level for the primordial and primary prevention of CV related mortality and morbidity [58]. This information can also be used to motivate young women to be physically active throughout the day by walking to workplace, using steps instead of a lift and taking opportunity to move around during working hours. This can be complimented by prescribing PA targets such as daily step counts and reduced sleeping hours that increase or preserve SMM, reduce FM through a number of mechanisms leading to CV benefits [60].

Current study used randomly selected disease-free healthy sample from the community and used central DXA to measure body composition, which can be considered the strengths of the study. However, cross-sectional nature of study, including only a single geographical area and low sample size may limit the generalizability of findings.

## Conclusions

This study reveals that some CV risk factors are associated with both FM and SMM among PrMW. However, no such associations were found among PMW. More studies should be done to reconfirm these results and if proven, this information should be used to inculcate healthy behavioral changes among middle aged women.

## Data Availability

The datasets used and/or analyzed during the current study are available from the corresponding author on reasonable request.

## Abbreviations

CVD: Cardiovascular disease
PMW: Postmenopausal women
PrMW: Premenopausal women
FM: Fat mass
SMM: Skeletal muscle mass
BMD: Bone mineral density
BMC: Bone mineral content
CVDR: Cardiovascular disease risk
STRAW: Stages of Reproductive Aging Workshop
NCD: Non-communicable diseases
TBFM: Total-body fat mass
TrFM: Trunkal fat mass
TBSMM: Total-body skeletal muscle mass
TBBMD: Total-body bone mineral density
TBBMC: Total-body bone mineral content
ASMM: Appendicular skeletal muscle mass
THBMD: Total hip bone mineral density
PA: Physical activity
IPAQ: International physical activity questionnaire
HDR: Hour dietary recall
BP: Blood pressure
SBP: Systolic blood pressure
DBP: Diastolic blood pressure
FBS: Fasting blood sugar
TC: Total cholesterol
TG: Triglycerides
HDL: High-density lipoproteins
LDL: Low-density lipoprotein
FRS: Framingham Risk Score
